# Genome-Wide Identification, Classification, Expression and Duplication Analysis of bZIP Family Genes in *Juglans regia* L.

**DOI:** 10.3390/ijms23115961

**Published:** 2022-05-25

**Authors:** Zhongrong Zhang, Shaowen Quan, Jianxin Niu, Caihua Guo, Chao Kang, Jinming Liu, Xing Yuan

**Affiliations:** 1 Department of Horticulture, College of Agriculture, Shihezi University, Shihezi 832003, China; zzr@stu.shzu.edu.cn (Z.Z.); qsw229@stu.shzu.edu.cn (S.Q.); gch202205@163.com (C.G.); kcshzu@163.com (C.K.); ljm20220526@163.com (J.L.); 18899533862@163.com (X.Y.); 2 Xinjiang Production and Construction Corps Key Laboratory of Special Fruits and Vegetables Cultivation Physiology and Germplasm Resources Utilization, Shihezi 832003, China

**Keywords:** *Juglans regia* L., bZIP gene family, evolutionary analyses, flower bud differentiation, expression pattern analysis

## Abstract

Basic leucine zipper (bZIP), a conserved transcription factor widely found in eukaryotes, has important regulatory roles in plant growth. To understand the information related to the bZIP gene family in walnut, 88 *JrbZIP* genes were identified at the genome-wide level and classified into 13 subfamilies (A, B, C, D, E, F, G, H, I, J, K, M, and S) using a bioinformatic approach. The number of exons in *Jr*bZIPs ranged from 1 to 12, the number of amino acids in *Jr*bZIP proteins ranged from 145 to 783, and the isoelectric point ranged from 4.85 to 10.05. The majority of *JrbZIP* genes were localized in the nucleus. The promoter prediction results indicated that the walnut *bZIP* gene contains a large number of light-responsive and jasmonate-responsive action elements. The 88 *JrbZIP* genes were involved in DNA binding and nucleus and RNA biosynthetic processes of three ontological categories, molecular functions, cellular components and biological processes. The codon preference analysis showed that the bZIP gene family has a stronger bias for AGA, AGG, UUG, GCU, GUU, and UCU than other codons. Moreover, the transcriptomic data showed that *JrbZIP* genes might play an important role in floral bud differentiation. The results of a protein interaction network map and kegg enrichment analysis indicated that *bZIP* genes were mainly involved in phytohormone signaling, anthocyanin synthesis and flowering regulation. qRT-PCR demonstrated the role of the bZIP gene family in floral bud differentiation. Co-expression network maps were constructed for 29 walnut *bZIP* genes and 6 flowering genes, and *JrCO* (a homolog of *AtCO*) was significantly correlated (*p* < 0.05) with 13 *JrbZIP* genes in the level of floral bud differentiation expression, including *JrbZIP31* (homolog of *AtFD*), and *JrLFY* was significantly and positively correlated with *JrbZIP10,11,51,59,67* (*p* < 0.05), and the above results suggest that bZIP family genes may act together with flowering genes to regulate flower bud differentiation in walnut. This study was the first genome-wide report of the walnut bZIP gene family, which could improve our understanding of walnut bZIP proteins and provide a solid foundation for future cloning and functional analyses of this gene family.

## 1. Introduction

Transcription factors are an important class of regulatory elements that can specifically bind to cis-acting elements that regulate genes and play important regulatory roles in many of the biological activities of plants [1]. The bZIP transcription factor consists of a basic region and a leucine zipper structure [2], of which the basic region is relatively conserved and consists of approximately 16 amino acid residues, which bind specifically to the DNA cis progenitor through the fixed nuclear localization signal structure N-(X)7-R/K [3]. The basic region binds specifically to DNA cis-elements with the core-binding sequence ACGT. Different bZIP proteins bind specifically to A-box, C-box or G-box, and some bind to non-retroviral sequences [4]. The leucine zipper region located at the C-terminus is closely linked to the basic region and acts mainly through the hydrophobic surface to form homo- or heterodimers of bZIP proteins [5].

The bZIP family of genes has a long evolutionary history, dating back to the early ancestors of green algae, and thus bZIPs are associated with a large number of functions. The Arabidopsis bZIP family contains 78 members. These bZIPs are classified into 13 groups (A-M) [3]. Group B (*bZIP17*, *bZIP28*, and *bZIP49*) and Group K (*bZIP60*) are important regulators of the endoplasmic reticulum (ER) stress response [6]. The group E member *bZIP34* is closely related to pollen germination and pollen tube growth [7], and group M contains only one member, *bZIP72*. Group I contain 12 members. *bZIP51* is considered to be a putative host factor in Agrobacterium-mediated T-DNA transfer [8]. Group D consists mainly of TGA transcription factors, named after their homologous TGACG DNA-binding motifs. Group G has five members (*GBF1-5*) that interact with two members of group H, *HY5*, and its homolog *HYL*, and are involved in the regulation of blue light-induced hypocotyl growth and in the regulation of chlorophyll and anthocyanin accumulation [9,10]. The S group consists of 17 intron-less bZIP members involved in seed maturation [11] and response to salt stress in roots [12], and in plant starvation signaling, members of group C can form a C/S1-bZIP network, suggesting that these isoforms are functionally interconnected [13]. Group A contains 13 members that are further split into four subgroups, mainly involved in the response to various abiotic stresses and the regulation of flowering, for example, *ABF1*, which have been found to function in the core part of ABA [14]. The genes are highly associated with flowering, and *FD* and *FDP*, classified as another A subgroup, are required for *FT* function [15]. Both genes are expressed in the apical bud and interact with *FT* in the nucleus to promote the transcription of key floral meristematic tissue characteristic genes, such as *APETALA1* [16].

The *bZIP* gene of Arabidopsis has been well studied. However, only a few *bZIP* genes in other plants have been studied. GhFD1 and GhFD2 promote early flowering in cotton by forming ternary complexes (FACs) with Gh14-3-3 and GhFT proteins [17]. The overexpression of Arabidopsis *FD* and *FDP* in rice resulted in reduced height and smaller rice spikes in transgenic rice [18]. The overexpression of *ZmbZIP4* in maize increases abscisic acid synthesis with an increase in the lateral root number [19]. *StbZIP61* can regulate salicylic acid biosynthesis by affecting the expression of *StICS1*, a key enzyme in SA biosynthesis [20]. Like *StbZIP61* in potato, sweet orange *CsbZIP40* acts by positively regulating SA biosynthesis [21]. In conclusion, the above studies suggest that the bZIP gene family is widely involved in plant growth and development.

Walnut (*Juglans regia* L.) is a woody plant of the genus Walnut in the family Jugladandaceae. There are more than 20 species of walnuts worldwide, distributed in parts of Asia, such as central Asia, west Asia, south Asia, and Europe [22]. Walnut production is increasing every year, the majority of which is produced in China, with the United States and France accounting for 30% and 11%, respectively [23]. Due to the low yield and poor quality, the economic benefits of the walnut industry are less obvious. The most important reason for this is that the number of male flowers is too high, leading to the consumption of a lot of nutrients and seriously affecting the differentiation and development of female flowers, which causes a yield reduction in walnuts in the following year. In this study, we analyzed the walnut bZIP gene family using bioinformatics methods and identified the number of walnut bZIP family members at the genome level. We explored the evolutionary relationships, subfamily classification, intron and exon structures, codon bias, GO and KEGG enrichment analyses, protein network relationships, and the expression patterns of the bZIP gene family in different developmental processes of different tissues in walnut. We provide a useful resource for future molecular breeding studies in regulating floral bud differentiation in walnut. 

## 2. Results

### 2.1. Identification, Chromosomal Localization and Physicochemical Property Analysis of bZIP Gene Family Members in Walnut

By similarity searches and structural domain determination, a total of 88 bZIP family members were identified in walnut and named *JrbZIP1*~*JrbZIP88*, and all of them contained the BRLZ domain (Figure S1). In addition, the conserved regions of the bZIP family members were extracted and the results showed that the bZIP structural domains all contained an N-X7-R/K-X9-L-X6-L-X6-L motif by multiple sequence alignment (Figure S2). The chromosome localization (Figure S4) showed that the 88 *Jr*bZIP gene family members were unevenly distributed on 16 chromosomes. Among them, chromosomes 8 and 11 had the largest number of *JrbZIP* genes, 11 each, and chromosome 2 had the fewest, with two. The physical and chemical properties of the walnut bZIP family members were analyzed, and the results showed that the theoretical isoelectric point (PI) of *Jr*bZIP ranged from 4.85 (*JrbZIP33*) to 10.05 (*JrbZIP83*), the relative molecular masses ranged from 16.37 KDa (*JrbZIP2*) to 84.52 KDa (*JrbZIP51*), and the amino acid length readings ranged from 145 aa (*JrbZIP2*) to 783 aa (*JrbZIP51*). Subcellular localization analysis revealed that all *Jr*bZIP proteins were located in the nucleus except for protein *JrbZIP41*, which was located in the cytoplasm and nucleus (Table S1).

### 2.2. Phylogenetic Analysis of Walnut bZIP Family Members

**Figure 1:**
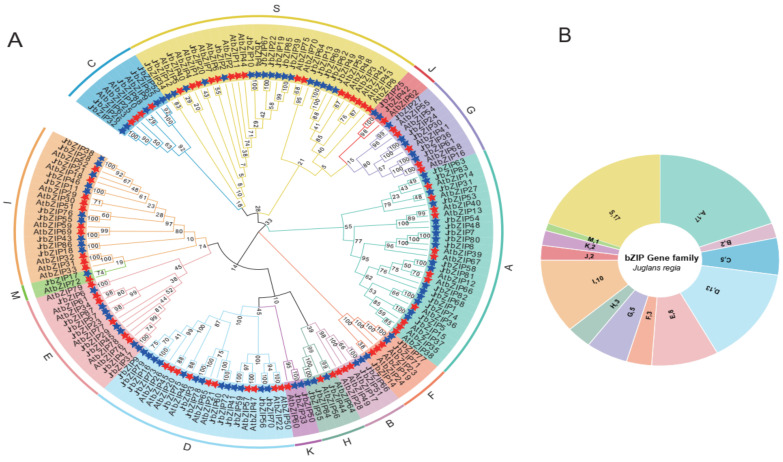
(**A**) Phylogenetic relationships among the identified bZIP proteins in *Arabidopsis thaliana* and *Juglans regia* L. The red stars indicate the *At*bZIP proteins, and the blue stars represent the *Jr*bZIP proteins. (**B**) The number of each group of the bZIP gene family of walnut.

To analyze the evolutionary relationship of the walnut bZIP transcription factors, the bZIP protein sequences of walnut (88 members) and Arabidopsis (75 members) were integrated and a phylogenetic tree was constructed. Referring to the grouping rules in Arabidopsis [3], walnut bZIP proteins were divided into 13 subgroups (Figure 1A). Among these 13 subgroups, group S contained the most members, 33 in total, including 17 in walnut and 16 in Arabidopsis, while group M included the fewest members, one in walnut and one in Arabidopsis (Figure 1B). *At*FD and *At*FDP constitute one of the four subgroups in Arabidopsis group A. In our study, *Jr*bZIP31, *Jr*bZIP53, *Jr*bZIP63, *Jr*bZIP83 and *At*FD (*At*bZIP14), and *At*FDP (*At*bZIP27) were clustered into one group. *At*bZIP72 was associated with JrbZIP17, and so were clustered into one group. Each branch contained bZIP protein members from both walnut and Arabidopsis, indicating that species differences did not cause the bZIP transcription factor family members to cluster separately.

### 2.3. Gene Structure and Motifs Analysis of JrbZIP Members

Motif analysis showed that a total of 20 motifs were identified in 88 walnut bzip protein sequences, and most of the members of the same subgroup contained the same motif composition and sequential arrangement (Figure S2A). For example, all members of group H contain motif 1 and motif 4; members of group C contain motif 1, motif 4 and motif 12. Moreover, we also found that some conserved motifs existed only in specific subgroups; for example, motif 10 existed only in subgroup E and motif 8 existed only in subgroup A. Gene structure and evolutionary tree analysis (Figure 1A and Figure S2B) showed that all 17 members of group S contained only one exon; all members of group I had four exons and three introns, and five of the 13 members of group D had 11 exons and 10 introns. For example, group S *JrbZIP12* contains three conserved motifs, motif 1, motif 4 and motif 12, and contains only one exon and no introns.

### 2.4. Cis-Elements Analysis in JrBZIPs Promoter Regions

**Figure 2:**
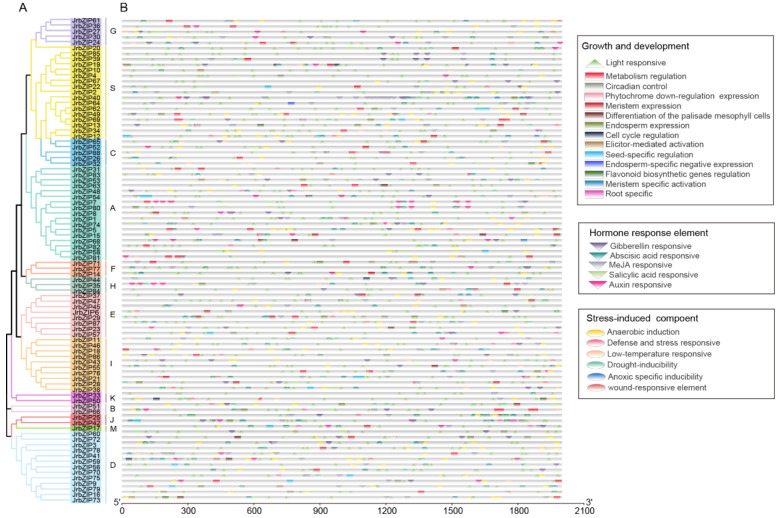
Phylogenetic relationships (**A**), and distribution of cis-elements in the promoter regions of *JrbZIPs* (**B**). The cis-acting elements were identified by PlantCARE using the upstream 2000 bp sequences of the *JrbZIPs*.

The promoter regions of the *JrbZIP* genes were analyzed using the Plantcare database. The cis-elements were divided into three main subgroups: plant hormones (such as auxin, jasmonic acid, salicylic acid and abscisic acid) and development related (such as light response, circadian rhythm control, meristem expression, endosperm expression, zein Protein, root-sperhythm control, meristem expression, endosperm expression, zein Protein, root-specific expression and cell cycle regulation), stress related (such as low-temperature, anaerobic induction, hypoxia, defense, stress and wound response) (Figure 2). Light-responsive elements are present in every walnut bZIP family gene, and jasmonic acid and anaerobic-inducible elements are commonly present in plant hormones and environmental factors. However, there are differences in different genes: *JrbZIP35* contains 5 light-responsive elements and 4 ABA-responsive elements, but *JrbZIP31* contains 15 light-responsive elements and 2 ABA-responsive elements, and there are no jasmonic acid-responsive elements in both of them. Flavonoid biosynthesis gene regulatory element only exists with *JrbZIP6,13,32,50,73*.

### 2.5. Chromosomal Distribution and Synteny Analysis of JrbZIPs

**Figure 3:**
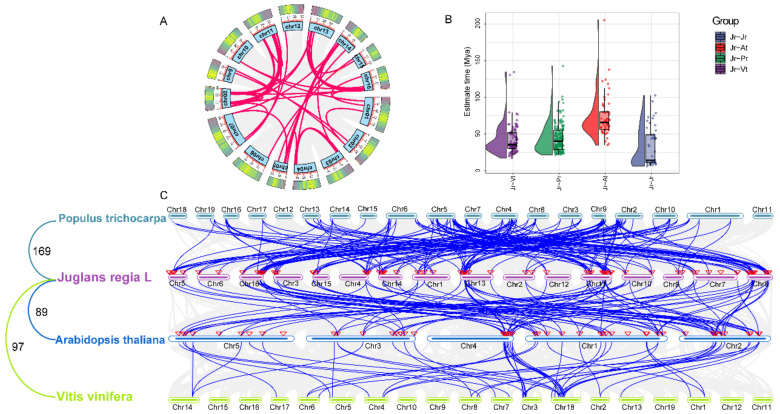
(**A**) Distribution and segmental duplication of *bZIP* genes in walnut. The blue panel showed the 16 chromosomes using a circle, red lines connecting homologous genes; (**B**,**C**) analysis of the Arabidopsis, Populus trichocarpa, grape, and walnut bZIP families. The gray lines in the background indicate the collinear blocks between walnut and other plant genomes, while the blue lines highlight the syntenic *bZIP* gene pairs. The chromosome number is shown at the top of every chromosome.

The synteny relationship between the bZIP gene family in walnut and three other species (*Populus trichocarpa*, *Arabidopsis thaliana* and *Vitis vinifera*) was further explored. In total, 169, 89 and 97 pairs of orthologous *bZIP* genes were detected in *Juglans regia* L. vs. *Populus trichocarpa*, *Juglans regia* L. vs. *Arabidopsis thaliana*, *Juglans regia* L. vs. *Vitis vinifera*, respectively. The divergence times of adjacent gene pairs were also calculated for the three species, showing that the replication event occurred approximately 17.38-205.27 million years ago (Mya). The divergence time of the bZIP family between Juglans regia L. and Arabidopsis thaliana (34.97–205.27 Mya) was greater than that between *Juglans regia* L. and the other two species, providing further evidence of a close relationship between Juglans regia L. and Vitis vinifera (17.38–134.45 Mya) (Figure 3B,C; Table S2). With the exception of individual bZIP family genes, the walnut bZIP family genes have a pair of multiple co-linearities with Arabidopsis bZIP family genes. For example, the *AtFD* gene has a covariate relationship with *JrbZIP31/53/63/83*. In addition, we analyzed the synteny relationship of the bZIP gene family within the walnut genome and identified a total of 1 pair of tandem duplication events (*JrbZIP1/JrbZIP74*) and 46 pairs of fragment duplication events, and calculated the substitution rate ratio Ka/Ks and differentiation time between these gene pairs by TBtools software. Ka/Ks ratios are all less than 1, indicating that bZIP family evolution is mainly determined by purifying selection. The Ks values were further used to calculate the divergence times of the 46 replicated gene pairs. The divergence times of these gene pairs ranged from 6.17 to 102.57 Mya. The earliest gene duplication event occurred at 102.57 Mya (Figure 3B; Table S2).

### 2.6. Analysis of Base Number and Codon Usage Bias of JrBZIPs

**Figure 4:**
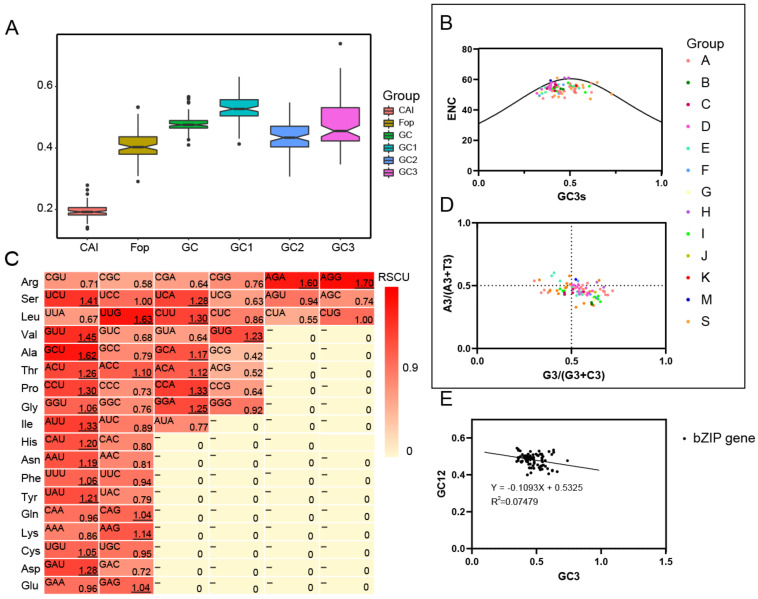
Analysis of base number and codon usage bias of *Jr*BZIPs. (**A**) The bZIP gene family codon uses bias parameters. (**B**) ENC plot analysis of bZIP gene family. (**C**) The frequency of synonymous codons in Walnut bZIP. The underlined data indicate that walnut *bZIP* gene uses this codon more frequently. (**D**) PR2-plot analysis of bZIP gene family PR2 plot. (**E**) Neutral mapping analysis of bZIP gene family.

The results showed that U3s and A3s of bZIP family were generally more abundant than G3s and C3s; the average content of GC was 48%, GC3s was 45%, and AT3s was 55%, indicating that bZIP family genes preferentially use synonymous codons ending in A/U. ENC ranged from 47.19 to 61, and 88 CDS had ENC values higher than 45, accounting for 100%, indicating that the bZIP gene family had a weak codon usage bias when encoding amino acids. CAI ranged from 0.137 to 0.279, with an average of 0.194, among which the *JrbZIP22* gene had the highest expression level, and Fop ranged from 0.291 to 0.533, with an average of 0.409 (Figure 4A; Table S3).

The ENC mapping results showed that the ENC values of all 88 genes of the bZIP family were lower than the standard curve and the distribution range was relatively concentrated, indicating that the codon bias of the 88 *bZIP* genes was influenced by more than just mutational pressure (Figure 4B). PR2 plot analysis showed that the values of A3/(A3 + T3) or G3/(G3 + C3) deviate from 0.5. the frequency of use of the bases U, A, G, and C at position 3 of the codon was different, which suggested that both mutation pressure and natural selection affect the codon preference of bZIP family genes (Figure 4D). The correlation between GC12 and GC3 reached a significant level (*p* < 0.05), indicating that the codon preference of the walnut bZIP family gene was mainly affected by mutation pressure (Figure 4E).

The CodonW1.4.2 software was used to calculate the relative codon usage of 59 codons of 88 genes in the bZIP family to measure the codon usage bias of these genes. A total of 27 codons were high-frequency codons, and their RSCU values were all greater than 1. Among them, four codons, AGA, AGG, GCU, and UUG, had RSCU values greater than 1.6, which were highly preferred. Among them, AGG was used the most frequently and had an RSCU value of 1.70, and had the strongest codon preference; GCG was used the least frequently with an RSCU value of 0.42, and had almost no codon preference (Figure 4C).

R was used to analyze parameters related to codon preference in the walnut bZIP gene family. The results indicate that GC3s showed a highly significant positive correlation with GC, FOP, G3s and C3S (*p* < 0.001), a highly significant negative correlation with U3s and A3s content (*p* < 0.001), and a significant negative correlation with GC12 (*p* < 0.05); CAI values showed a highly significant positive correlation with C3s and FOP (*p* < 0.01), and a significant negative correlation with A3s (*p* < 0.01) (Figure S5).

### 2.7. Functional Annotations of JrbZIP Genes

**Figure 5:**
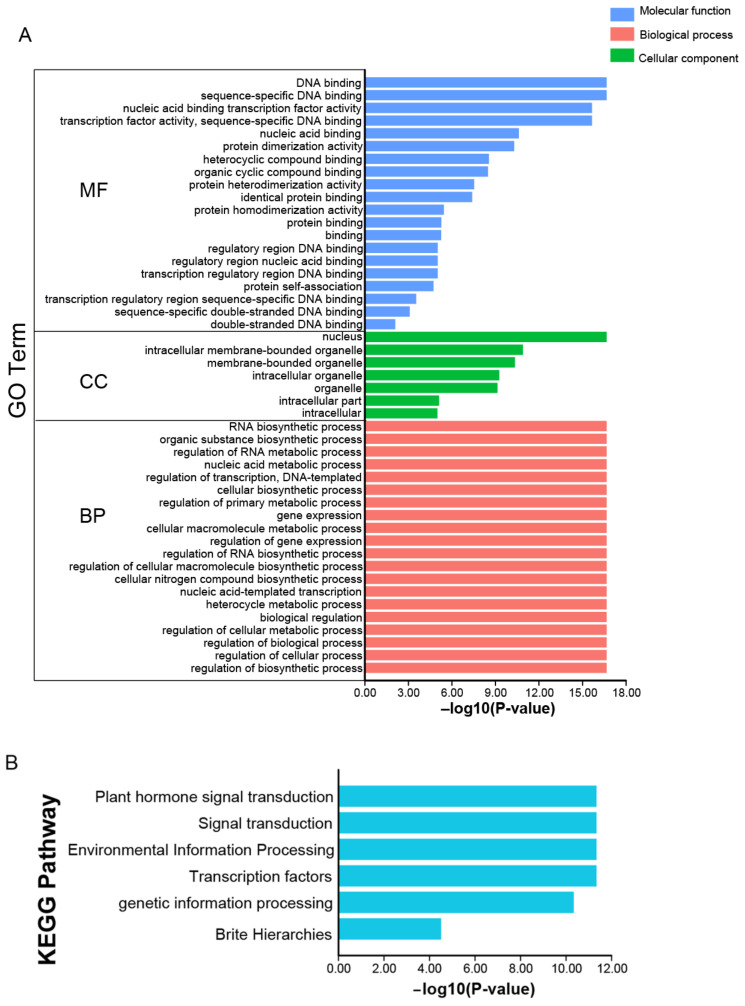
GO and KEGG enrichment of the *JrbZIP* genes. (**A**) GO enrichment of the JrbZIP genes. According to the secondary terminology, the GO annotation results are divided into three ontological categories and distinguished by different colors. (**B**) KEGG enrichment of the JrbZIP genes KEGG annotation results are represented by a blue bar graph.

GO and KEGG enrichment analysis facilitates a better understanding of the life activities and metabolic processes involved in walnut bZIP gene family members. According to GO enrichment analysis, the 88 *JrBZIP* genes were involved in DNA binding, nucleic acid binding, protein binding, nucleus, intracellular membrane-bounded organelle and the RNA biosynthetic process of three ontological categories of molecular functions, cellular components and biological processes (Figure 5A). KEGG analysis revealed that the *bZIP* gene of walnut plays a major role in signaling in metabolic pathways, including hormone signaling, signal transduction, and environmental information processing, etc. (Figure 5B).

### 2.8. Expression Profile Analysis of Walnut bZIP Genes

To further reveal the expression pattern of bZIP family genes in walnut, we used transcriptome data from female flower buds and leaf buds of Juglans regia cv. Xinxin No.2, as well as transcriptome data from female and male flower buds of Juglans regia L. cv. Wen185. For female and male flower buds, we collected female flower buds from three periods and male flower buds from three periods.

**Figure 6:**
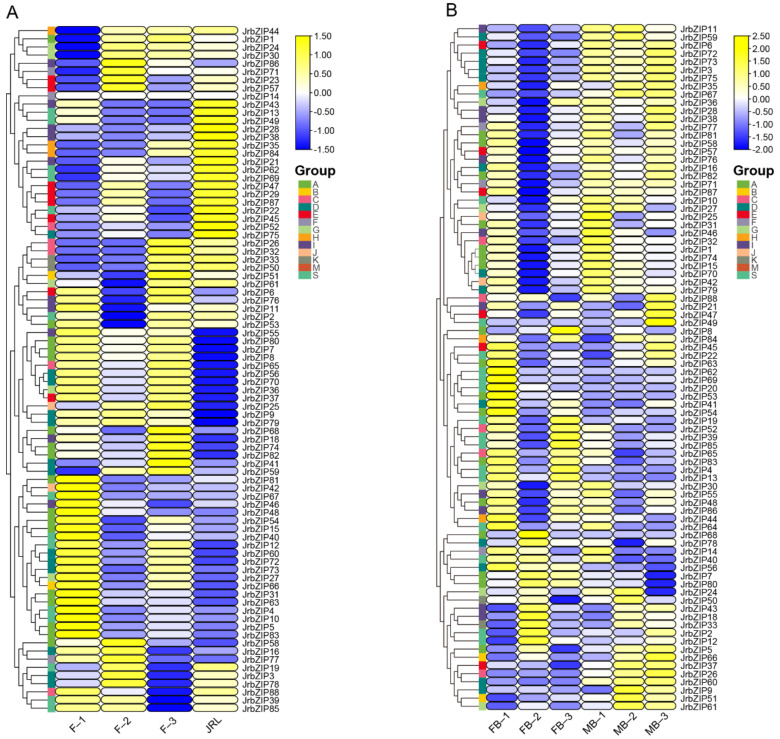
Expression profiles of candidate *JrbZIP* genes in tissues or different developmental stages. (**A**). Heat map of *JrbZIP* gene in three periods of female flower bud development (F–1, F–2 and F–3) and one period of leaf bud development (JRL; leaf buds of the same period as F–2). (**B**). Heatmap of *JrbZIP* genes expressed differently in three development periods of female and male flower buds (FB/MB–1, FB/MB–2, and FB/MB–3).

Cluster analysis showed that the walnut bZIP transcription factor family was divided into seven classes in “XinxinNo.2”. The relative expressions of twelve *JrbZIP* genes (i.e., *bZIP7/8/9/25/36/37/55/56/65/70/79/80*) were gathered together due to their low expression in JRL and low expression in the early and late stages of floral bud differentiation. In contrast, 17 *JrbZIP* genes (i.e., *bZIP13/21/22/28/29/35/38/43/45/47/52/62/69/72/84/89*) were gathered together due to their low expression in the early and late stages of floral bud differentiation and high expression in JRL, and 20 *JrbZIP* were highly expressed during the F_1 period, especially *JrbZIP4/5/10/31/42/63/83* (Figure 6A and Figure S4A). The bZIP gene family in “Wen185” was clustered into six classes. The relative expressions of twelve *bZIP* genes (i.e., *bZIP3/6/11/28/35/36/38/59/67/72/73/75*) were clustered together due to their low expression in FB_1, FB_2, FB_3 and high expression in MB_1, MB_2, MB_3. In addition, we observed a high expression of *JrbZIP1/10/15/25/27/32/42/46/70/79* at the FB-1 and MB-1 periods and low expression at other periods of floral bud differentiation, suggesting that they may be associated with initial floral bud differentiation (Figure 6B and Figure S4B).

### 2.9. Analysis of JrbZIP Protein–Protein Interaction

**Figure 7:**
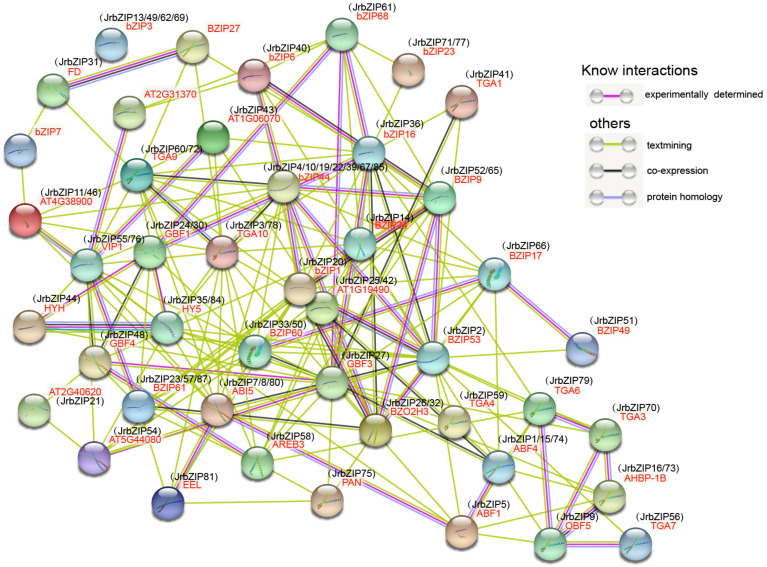
Predicted protein–protein interaction network of *Jr*bZIP protein. The network nodes represent proteins. The 3D structure of the protein is shown inside the nodes, and the line color indicates the type of interaction evidence.

Based on orthoVenn2 analysis, evolutionary analysis, and KEGG pathway analysis, we constructed a protein interaction network relationship map of 88 *Jr*bZIP proteins in walnut (Figure 7 and Figure S5). *Jr*bZIP proteins were mainly classified into three major classes: *ABI5* (*Jr*bZIP7/8/80), involved in plant hormone signaling, AREB3 (*Jr*bZIP58), AT5G44080 (*Jr*bZIP54), PAN (*Jr*bZIP75), TGA1 (*Jr*bZIP41), TGA3 (*Jr*bZIP70), TGA4 (*Jr*bZIP59), TGA10 (*Jr*bZIP3/78), TGA6 (*Jr*bZIP79), TGA7 (*Jr*bZIP56) and TGA9 (*Jr*bZIP60/72), HY5 (*Jr*bZIP35/84) involved in cryptochrome signaling, and FD (*Jr*bZIP31), which promotes excessive flowering.

### 2.10. Analysis of the Role of JrbZIPs in Flowering

**Figure 8:**
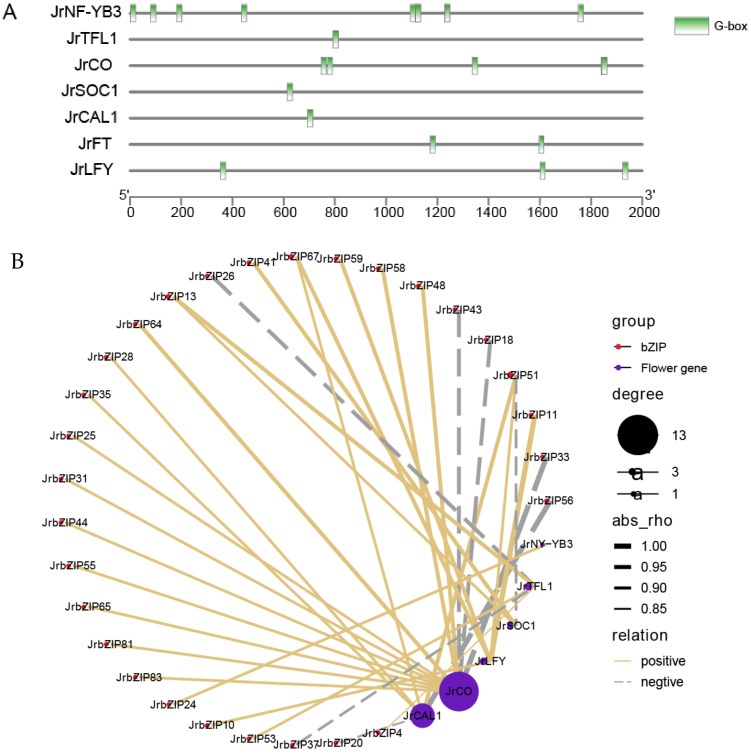
(**A**) The promoter sequences (2000 bp) of 7 flower genes were analyzed. The elements were identified as follows: green for the G-box element. (**B**) Co-expression network of 29 *bZIP* genes and 6 flowering genes in walnut. Co-expression network of 29 *bZIP* genes from walnut with six flowering genes. The genes (nodes) in red and blue ovals represent probes (flowering genes) and targets (*bZIP* genes), respectively. Co-expression values are based on Pearson’s correlation coefficient ≥0.8, with a *p* value cutoff at 0.05. The lines in yellow and black indicate the positive and negative correlations between the two nodes, respectively.

Flower bud differentiation is an important process in plant flowering, and to further investigate the common role of walnut *bZIP* genes and flowering genes in walnut flowering bud differentiation, we constructed a co-expression network map between six flowering genes and *JrbZIP* genes (Figure 8B). All of the flowering genes used to construct the network map contained G-box-acting elements, with *JrNY-YB3* containing seven G-box-acting elements, the most of all flowering genes (Figure 8A). Finally, combined with the correlation coefficients, we constructed a total of 33 network maps between *JrbZIP* genes and six flowering genes. Among them, *JrCO* was positively correlated with 10 *JrbZIP* genes, and the other four flowering genes (*JrCAL1*, *JrTFL1*, *JrSOC1*, *JrNF-YB3*, and *JrLFY*) were positively and negatively correlated with 15 and 8 genes, respectively (Figure 8B).

### 2.11. Verification of JrbZIP Expression Pattern by qRT-PCR

In total, 11 genes (*JrbZIP1/31/35/48/53/60/63/83/84, JrCAL1/LFY*) were selected for RT-qPCR analysis, according to *JrbZIP* gene heat map analysis (Figure 6) and previous studies [24]. 

**Figure 9:**
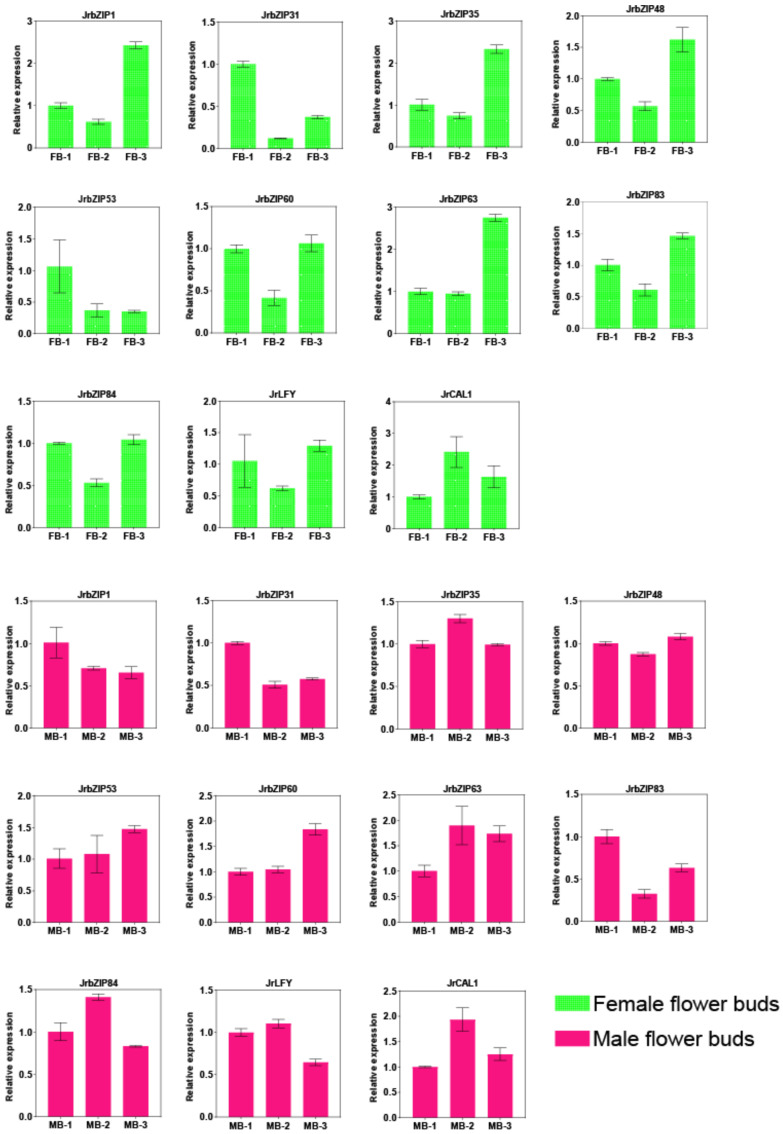
qRT-PCR analysis of *JrbZIP* genes in different tissues and development periods of flower buds. FB-1, FB-2, FB-3, MB-1, MB-2, and MB-3 represent the different developmental periods in female (green bar chart) and male (red bar chart) flower buds, respectively.

The experimental results showed that in female flower buds, eight *JrbZIP* genes (*JrbZIP1/31/35/48/60/83/84* and *JrLFY*) were highly expressed in early and late bud differentiation, and *JrCAL1* was highly expressed in mid bud differentiation, while *JrbZIP53* was highly expressed only in early bud differentiation. In male flower buds, *JrbZIP48* and *JrbZIP83* were lowly expressed at mid floral bud differentiation, whereas *JrbZIP35*, *JrbZIP84* and *JrCAL1* were highly expressed at mid floral bud differentiation; *JrbZIP1* was highly expressed at early floral bud differentiation, whereas *JrbZIP53* and *JrbZIP60* were lowly expressed at early floral bud differentiation. In total, 11 *JrbZIP* genes were highly expressed in the expression of 11 *JrbZIP* genes in female and male flower buds, which is generally consistent with the transcriptome sequencing results (Figure 9).

## 3. Discussion

The bZIP transcription factors are widely present in plant and play an important role in regulating growth and development as well as in response to stresses. However, systematic studies on the walnut bZIP gene family have not been reported due to the relatively slow research on nut fruit trees. 

### 3.1. Identification of bZIP Genes

Our genomic survey identified 88 members in the walnut bZIP TF family. It was greater than that of Arabidopsis [3] (n = 75), grapevine (n = 55) [25], tomato (n = 69) [26], cucumber (n = 64) [27], and Chinese jujube (n = 45) [28], but less than soybean (n = 131) [29], Chinese cabbage (n = 247) [30] and maize (n = 125) [31]. The number of *bZIP* genes between species may be related to gene duplication events and genome size [32]. 

### 3.2. bZIP Genes Clustering and Evolutionary Analysis

Phylogenetic analysis showed that *Jr*bZIPs could be divided into 13 subfamilies, and the results were similar to those of Arabidopsis, rice and poplar [33]. We analyzed the chromosomal locations of 88 identified bZIP family members and found that the *JrbZIP* genes were mainly distributed at both ends of chromosomes 1–16, which was similar to the results in *Cucumis sativus* [27] and sweet potato [34]. In walnut *bZIP* genes, there were 46 pairs of segmental duplication genes, and gene segmental duplication is an important factor for gene family generation. In total, 58% of the soybean bZIP genes undergo the fragment duplication phenomenon [29]. There were 169 pairs of nine covariates in the *bZIP* genes of walnut and Populus trichocarpa, and 89 pairs of genes with Arabidopsis, indicating that walnut was more closely related to Populus trichocarpa than to Arabidopsis. In addition, collinearity and selection pressure analysis indicated bZIP gene family expansion in walnut with purifying selection pressure. The most recent gene duplication event for most bZIP family genes occurred 10–40 million years ago (Table S2). The results of the bZIP codon bias analysis in walnut were similar to those reported in the walnut TALE gene family, with the JrbZIPs preferring codons ending in A/U [35]. If the codon bias of the bZIP family gene is completely affected by mutation pressure, the frequency of the purine base and pyrimidine base should be equal; that is, the frequency of the A base and T base should be equal, and the frequency of the G base and C base should be equal. This suggests that both mutation pressure and natural selection affect the codon preference of bZIP family genes.

### 3.3. Structural Diversity of bZIP Genes

The walnut bZIP gene family is highly conserved and all members contain motif 1, and the conserved motifs of related proteins are more similar to the gene structure. Each gene of the walnut bZIP transcription factor family contains introns and exons of different lengths and numbers, with the gene length ranging from 145 to 783 amino acids and the maximum number of introns being 13. According to previous studies, *bZIP* genes without introns are commonly found in bZIP family genes of various crops, such as rice and walnut, where 15.3% and 19.31% of bZIP genes have no introns, respectively. There were 19 members of the S group in poplar, and 18 members have only one exon and contain no introns [33]. The above results indicate that the gene structure of the S subgroup of the bZIP gene family is highly conserved in different species.

### 3.4. Specific Expression Patterns of bZIP Genes

Many studies have shown that the basic leucine zipper (bZIP) family of transcription factors was extensively involved in plant growth and developmental processes [7,36,37]. In our study, the expression levels of the *JrbZIP* gene were observed in ten developmental periods in two walnut varieties, indicating that *JrbZIP* is involved in regulating the growth and development of walnut (Figure 6). Apple bZIP family S-group genes, *MdbZIP39* and *MdbZIP72,* have significantly higher transcript levels with fruit development [38]. In our study, S-group genes were highly expressed in early F-1 and leaf buds of “Xinxin No.2” walnut, such as *JrbZIP13* and *JrbZIP49*, which were highly expressed in leaf buds. *JrbZIP12* was also highly expressed in early female flower bud differentiation. In addition, in “wen 185” male and female flower buds, group S genes were highly expressed mainly in early female flower bud differentiation, such as *JrbZIP20*, *JrbZIP62*, and *JrbZIP69*, which were highly expressed in the FB-1 period. The grapevine bZIP family gene *VvbZIP19* is a homolog of Arabidopsis *AtABI5* and is expressed mainly in seeds [25]. The group A member *PmABF7* (with the highest homology to Arabidopsis *AtABI5*) was highly expressed in buds and fruits in Prunus mume [39]. In addition, *AtABI5* was involved in the regulation of flowering through direct action on FLC transcription [15,40]. Here, we examined the expression of their orthologs, *JrbZIP7*, *JrbZIP8*, and *JrbZIP80*, which are mainly expressed at the early stage of “Xinxin No.2” female bud development and at the middle of “Wen185” female bud development (F-1, FB-2), but the mechanism of regulation of this expression remains unclear. *AtHY5* (*JrbZIP35/84*) plays an important role in the accumulation of anthocyanins, while its homologs, *JrbZIP35* and *JrbZIP84*, were highly expressed at later stages of leaf bud and male flower bud development. Homologs of Arabidopsis *FD* and *FDP* in rice can interact with *OsFT, Os14-3-3* to form a ternary complex controlling flowering in rice [41]. The overexpression of *PtFD1* in poplar resulted in the early flowering of juvenile plants under a long-day (LD) photoperiod [42]. The homolog of *AtFD* in grapes (*VvbZIP49*) was significantly more expressed in leaves and flower buds than in other tissues. Therefore, we detected the expression of its orthologs, *JrbZIP31*. The expression in the early stages of male and female flower bud development in walnut (F-1, M-1) was higher than that in leaf buds, suggesting that *FD* may be involved in the early stages of flower bud differentiation (Figure 5A,B and Figure 6).Previous studies found that *FcLFY* was involved in the process of floral bud differentiation and increased the expression of floral meristematic tissue and floral organ genes by overexpressing *FcLFY*, thus regulating flowering and appearing as an early flowering phenomenon [24]. In our study, a co-expression network of the *bZIP* gene family and seven flowering genes was constructed, and surprisingly, the correlation between the flowering vegetative gene *FT* and *bZIP* genes was low, while *JrCO* was significantly correlated (*p* < 0.05) with 13 *bZIP* genes, including *JrbZIP31* (a homolog of *AtFD*), and *JrLFY* was significantly positively correlated (*p* < 0.05) with *JrbZIP10, 11, 51, 59,* and *67;* the above results suggest that bZIP family genes may act together with flowering genes to regulate flower bud differentiation.

## 4. Materials and Methods

### 4.1. Plant Materials

To investigate the expression pattern of walnut bZIP family in flower bud differentiation, male and female flower buds of early fruiting walnut variety *Juglans regia* L. *cv. Wen185* were collected from 2 June to 10 June 2021, from the same walnut orchard in Aksu region, South Xinjiang Uygur Autonomous Region, China. Each sample was pooled from 3 buds, and 3 biological repeats were performed, for a total of 9 buds for each stage of the floral transition. The samples were wrapped in tin foil, quickly frozen in liquid nitrogen, and stored at −80 °C.

### 4.2. Database Search for bZIP Members in Juglans regia L.

Walnut whole genome data were downloaded from the NCBI database (http://www.ncbi.nlm.nih.gov/, accessed on 20 April 2021). The hidden Markov model files (PF00170, PF03131, PF07716, and CPF12498) of bZIP transcription factors were downloaded from the pfam database (http://pfam.xfam.org/, accessed on 26 April 2021) [43], and the above hidden Markov model files were used as search criteria. program in hmmer3.0 software to search for walnut protein sequences (E ≤ 1 × 10^−10^), and the obtained results were de-duplicated, and the results were extracted by SMART (http://smart.embl-heidelberg.de/, accessed on 26 April 2021) and NCBI-CDD (https://www.ncbi.nlm.nih.gov/Struc-ture/cdd/wrpsb.cgi, accessed on 27 April 2021) databases for further identification and screening, and finally the walnut bZIP family protein sequences were obtained [44]. Finally, the isoelectric point and molecular weight of walnut bZIP protein sequences were analyzed using the online tool ProtParam (https://www.expasy.org/protparam/, accessed on 27 April 2021) [45]. The online software Plant-mPLoc 2.0 (http://www.csbio.sjtu.edu.cn/bioinf/plant/, accessed on 28 April 2021) was used to predict and analyze the subcellular localization of walnut bZIP proteins [46].

### 4.3. Phylogenetic Analysis and Classification

The *Arabidopsis thaliana* bZIP protein sequences were downloaded from the TAIR database (https://www.arabidopsis.org/, accessed on 26 July 2021). We performed a multiple alignment of the 88 full-length *Jr*bZIPs and 75 full-length *At*bZIPs using ClustalW. We used the alignment result to construct a phylogenetic tree using the neighbor-joining method of MEGA7 software (http://www.megasoftware.net/, accessed on 15 March 2020). We created the phylogenetic tree using the following parameters: Poisson correction, pairwise deletion, and a bootstrap value of 1000 [47]. The resulting phylogenetic tree was further processed with the online tool iTOL (https://itol.embl.de/, accessed on 28 July 2021).

### 4.4. Sequence Analysis

GSDS v2.0 (http://gsds.cbi.pku.edu.cn/, accessed on 26 July 2021) was used to predict the inline-exon structure of the *bZIP* gene family in walnut [48]. Use MEME (http://meme-suite.org/tools/meme, accessed on 27 April 2021) to analyze the motif composition of JrbZIP proteins, motif size setting: 6–50, number setting: 20, the rest of parameters use default values, the results were visualized using the program “Gene Structure View (Advanced)” of the software TBtools [49,50]. From the NCBI Walnut Whole Genome Database (ftp://ftp.ncbi.nlm.nih.gov/genomes/refseq/plant/Juglans_regia/latest_assembly_versions/GCF_001411555.2_Walnut_2.0/, accessed on 27 April 2021) for each walnut *bZIP* gene promoter region (2000 bp upstream). The type, number and function of the cis-acting elements of the walnut bZIP gene promoter were analyzed using the PlantCARE (http://bioinformatics.psb.ugent.be/webtools/plantcare/html/, accessed on 28 July 2021) website [51]. Finally, the results were visualized using the program “Simple Biosequence Viewer” of the software TBtools [50].

### 4.5. Chromosomal Distribution and Synteny Analysis of JrbZIPs

Synteny relationship between walnut and the other three species (Arabidopsis, Populus, and grape) was sought using MCScanX [52]. Chromosomal localization and rates of synonymous substitutions (Ks), nonsynonymous substitutions (Ka), and evolutionary rates (Ka/Ks ratio) of the walnut *bZIP* gene family were determined using the TBtools software program “Gene Location Visualize from GTF/GFF” and the program “Simple Ka/Ks Calculator (NG)” [50]. In our studies, duplication events (t) were calculated according to the Ks value (t = Ks/2λ, λ = 1.5 × 10^−8^) [53,54].

### 4.6. Analysis of Codon Usage Pattern of JrbZIP Genes

Codonw1.4.2 software (http://codonw.sourceforge.net, accessed on 15 March 2022) [55] was used to analyze the codon usage characteristics of the walnut bZIP family of genes. Frequency of Optimal Codons (FOP), codon adaptation index CAI, overall GC content of codons, and GC content of codons coding for the same amino acid at position 3 (GC3S) were used to measure the base composition preference. The effective number of codon (ENC), GC1, GC2, GC3 of walnut bZIP family genes was also calculated using the CUSP and CHIP online programs in EMBOSS (http://embossgui.sourceforge.net/demo/, accessed on 16 March 2022) to evaluate the codon usage preference.

### 4.7. GO and KEGG Enrichment Analyses of JrbZIP Genes

GO and KEGG enrichment analyses were performed using the eggnog-mapper [56] tools for data analysis. Finally, we import the generated files into the “Enrichment Bar Plot” of TBtools software for visualization [50].

### 4.8. Expression Pattern Analysis of JrbZIP Genes

To investigate the expression pattern of the walnut *bZIP* gene family in leaf buds and flower buds, The expression data of “Xinxin No.2” leaf buds (JRL) and flower buds (F-1, F-2, and F-3) were obtained from previous transcriptome data [57], while the expression data of "Wen 185” male and female flower buds at different stages of differentiation and the quantification method were referred to Guo et al [35]. Briefly, FPKM values for different walnut bud differentiation were preprocessed with log2 and then plotted using the “HeatMap” program in TBtools software [50].

### 4.9. Protein Interaction Analysis Network of JrbZIP Protein

Arabidopsis bZIP protein with highest homology to walnut bZIP protein obtained by OrthoVenn2 online software (https://orthovenn2.bioinfotoolkits.net/home, accessed on 25 April 2022). The Arabidopsis *bZIP* gene was then used as the seed sequence that the specific parameters were E-value: 1 × 10^2^, Inflation value: 1.5, and finally the homologous protein of Arabidopsis thaliana in walnut was obtained. and then STRING (https://string-db.org, accessed on 14 April 2022) was used to map the bZIP protein interaction network [58]. 

### 4.10. Quantitative Real-Time PCR

Nine *JrbZIP* and two flowering genes that were differentially expressed in three periods of female and male flower buds were selected for qRT-PCR validation analysis. The walnut 18S gene was used as the internal reference gene for the *JrbZIP* gene [59]. Primers for 11 genes were designed on Primer 5 and subsequently handed over to Shanghai Biotech Biotech for primer synthesis. The primer sequences are detailed in Table S7. The level of gene expression was detected by qRT-PCR with the reaction system and conditions described by Quan et al. [57]. 

## 5. Conclusions

In our study, we identified a total of 88 *JrbZIP* genes genome-wide in walnut, with isoelectric points ranging from 4.85 (*JrbZIP33*) to 10.05 (*JrbZIP83*), molecular weights ranging from 16.37 KDa (*JrbZIP2*) to 84.52 KDa (*JrbZIP51*), and amino acid numbers ranging from 145 to 783. The 88 *JrbZIP* genes were mainly distributed at both ends of walnut chromosome 16, with the number of introns ranging from 1 to 13 and the number of motifs being between 1 and 9. The promoter predictions indicated that the light-responsive elements were widely present in the walnut bZIP family and that *JrbZIP* codon preference was weak, with mutational pressure and natural selection combining to influence codon preference in the walnut bZIP family. From protein interaction network analysis, as well as GO and KEGG enrichment analyses, the co-expression network and real-time fluorescence PCR results suggest that the bZIP gene family may play a joint role with flowering genes in walnut flower bud differentiation.

## Data Availability

All data in this study can be found in public databases and supplements, as described in the Section 4.

## Supplementary Materials

The following supporting information can be downloaded at https://www.mdpi.com/article/10.3390/ijms23115961/s1.
