# Supplementary material for: Genome-Wide Identification, Classification, Expression and Duplication Analysis of bZIP Family Genes in Juglans regia L
Source: Int J Mol Sci. 2022 May 25;23(11):5961. doi: 10.3390/ijms23115961 (PMC9180593; doi:10.3390/ijms23115961)
Supplement: Supplementary file 1 [file ijms-23-05961-s001.zip › Supplementary Information.pdf]

3.1 Identification and characterization of the bZIP transcription factor family in walnut

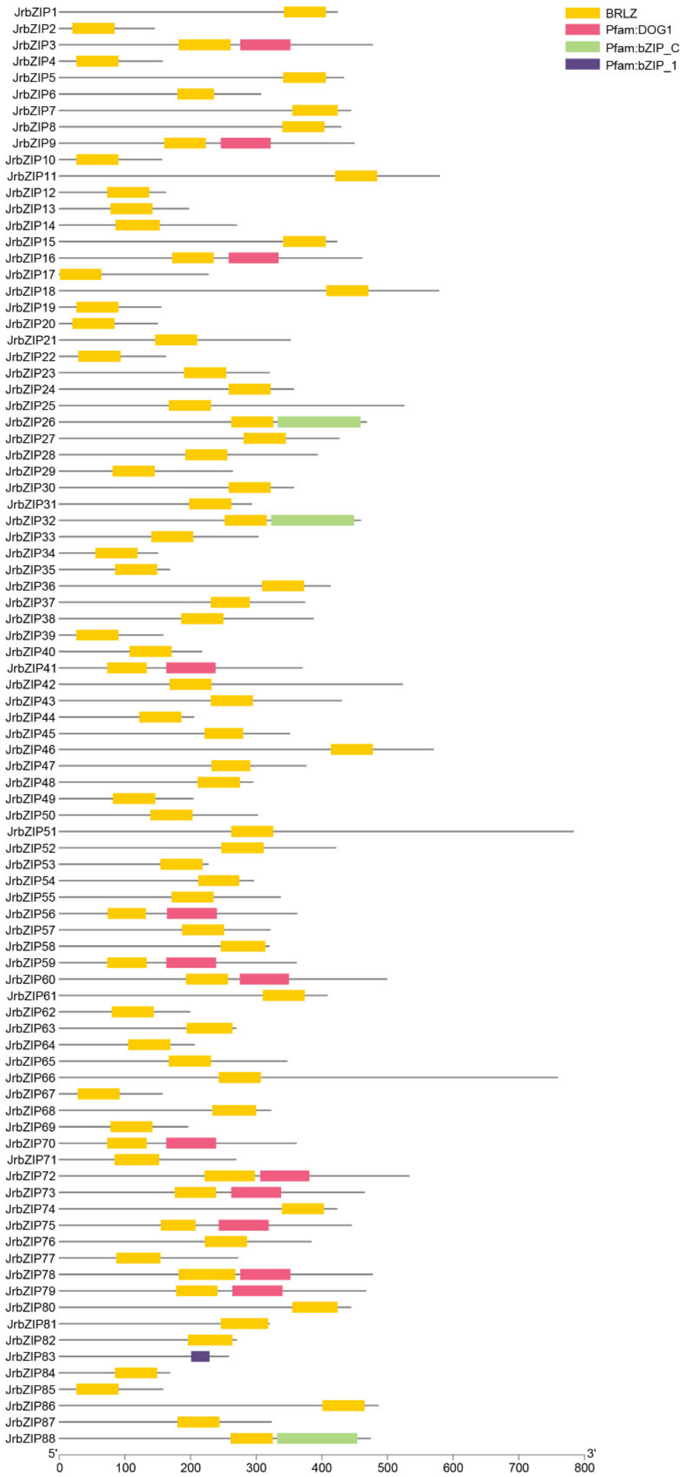

Figure S1. The main domain of walnut bZIP protein sequence.

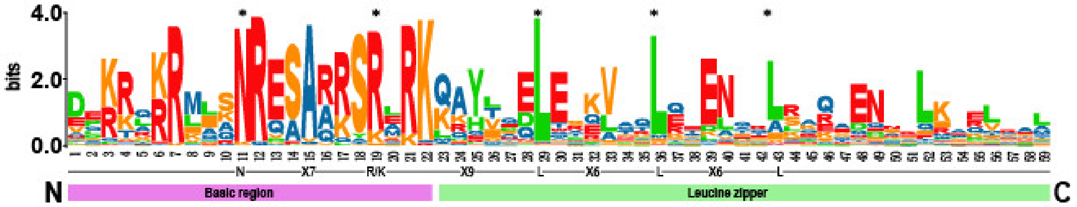

Figure S2. Visualization of multiple sequence alignment of the walnut bZIP family DNA binding domains. The total height of the letter piles at each position indicates the conservation of the sequence at that position (measured in bits).

3.3 Gene structure and characterization of conserved bZIP motifs from Walnut

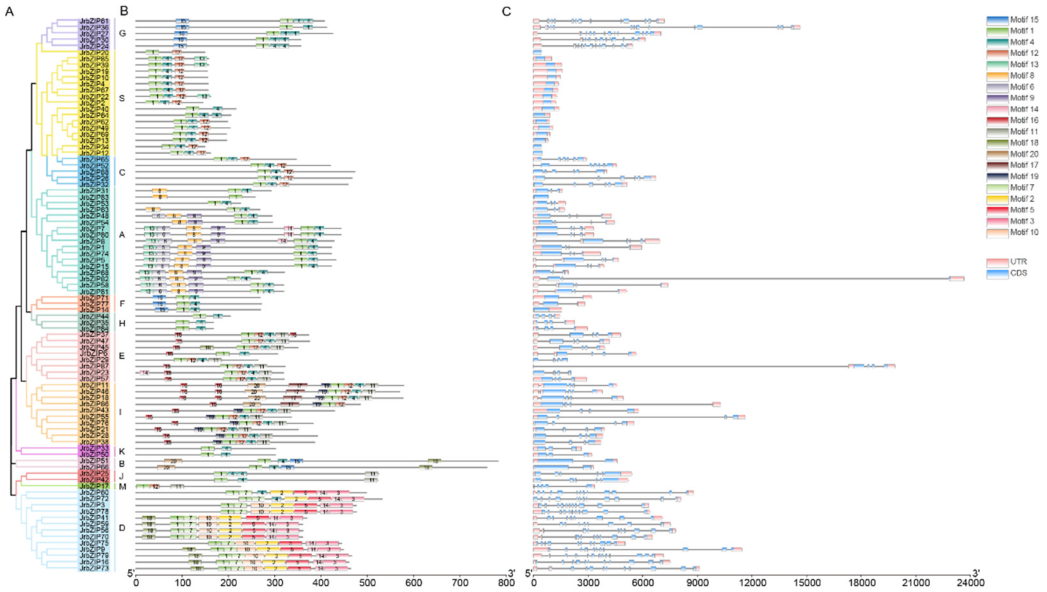

Figure S3. Phylogenetic relationships (**A**), motif compositions(**B**), and gene structures(**C**) of bZIPs in walnut. Gene structure dynamics of JrbZIPs were predicted with the GSDS software. The exons are represented by green boxes and the introns are indicated by black lines. The conserved motifswere scanned with MEME. Different motifs are represented by various colored boxes.

3.5 Chromosomal distribution and synteny analysis of JrbZIPs

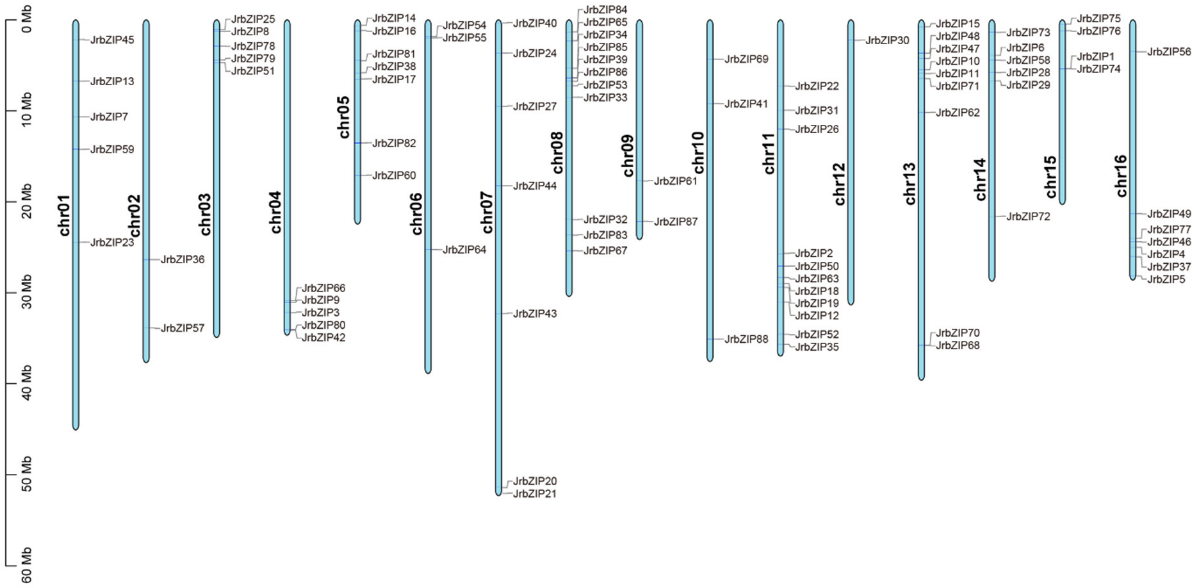

Figure S4. Chromosomal locations for JrbZIP gene family.

3.6 Analysis of base number and codon usage bias of JrBZIPs

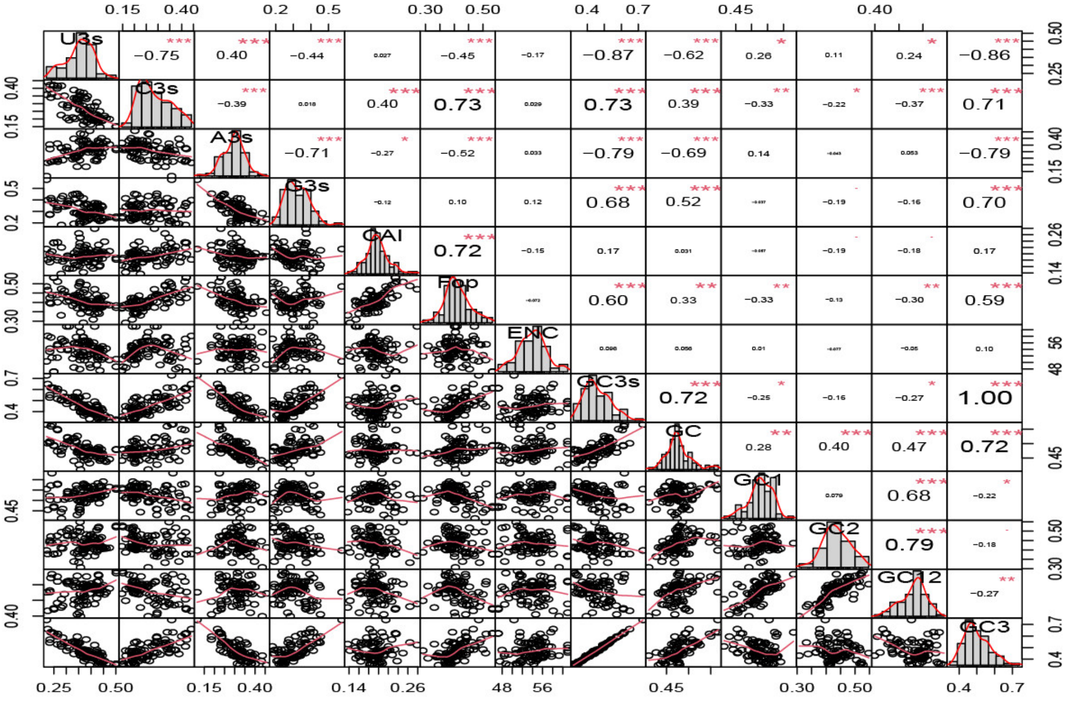

Figure S5. Correlation analysis of codon preference parameters of bZIP family genes notes: “ . ”、“*”、 “**” 、“***”Significant correlation was found at 0.1, 0.05,0.01and 0.001 levels (bilateral).
